# Music preferences, listening purposes, perfectionism and substance use in Egyptian college students: a cross-sectional study

**DOI:** 10.1186/s40359-026-04219-9

**Published:** 2026-03-26

**Authors:** NourAllah Ahmed, Mohamed Ahmed, Brycen Gillipsie, Farah Sharaf, Kamilia Salah, Lama Eissa, Reham Abdrabou, Retag Essayed, Reem Yasser, Yasmeen Zaky, Moustafa Sayed

**Affiliations:** 1 Center of Drug Research and Development (CDRD), Faculty of Pharmacy, The British University in Egypt, El Shorouk, Cairo Egypt; 2 Clinical Pharmacy Practice Department, Faculty of Pharmacy, The British University in Egypt, El Shorouk, Cairo Egypt; 3 School of Pharmacy, Marshall University, Huntington, WV USA

**Keywords:** Music preference, Egyptian folk music, Rap music, Substance use, Perfectionism, Listening context, Music psychology

## Abstract

**Background:**

Music plays an important role in psychological experiences, social interaction, and identity development, particularly among youth and young adults. This study examined associations between music preferences, listening contexts, and purposes with psychological traits, including perfectionism and substance use tendencies, among Egyptian college students.

**Methods:**

A cross-sectional survey was conducted among 400 Egyptian students aged 18–24. Data were collected via face-to-face and online questionnaires, which assessed music listening behaviours (based on a revised Music Preference Questionnaire), drug use tendencies, and perfectionism using the Big Three Perfectionism Scale (BTPS). Exploratory bivariate analyses were performed using chi-square tests and independent t-tests to examine group differences and associations.

**Results:**

Rap music preference was significantly more common among male students (*p* < 0.001) and, when listened to in social contexts such as parties or peer gatherings, was associated with higher substance use tendency scores (*p* = 0.002). Preference for Egyptian folk music was also associated with higher substance use tendency (*p* < 0.001), whereas classical Arab music preference was associated with lower substance use tendency (*p* = 0.03). Male students scored significantly higher than females in socially prescribed perfectionism (*p* = 0.028) and across subcomponents of narcissistic perfectionism, including entitlement, hypercriticism, grandiosity, and other-oriented expectations.

**Conclusions:**

Music preferences and listening contexts were associated with distinct psychological and behavioural patterns among Egyptian college students. Certain music genres and social listening contexts showed consistent associations with substance use tendency, while gender differences were observed in perfectionism dimensions. These findings underscore the relevance of music-related behaviours as correlates of psychological traits and support further longitudinal research to clarify directionality and underlying mechanisms.

## Background

Music and its influence on humans have been of great interest, and for decades there has been a growing interest regarding the psychological effects of music on humans, especially in social contexts [1]. Researchers found that there is a complex relationship between music and the human brain, from how individuals cognitively and emotionally process music to its influence on emotional experience and behavioural responses [2]. Music preferences were found to influence certain behaviours in adolescents and young adults and help in shaping their identity [3]. Accordingly, examining associations between music engagement and psychological traits such as aggression, substance use tendencies, and perfectionism among university students is particularly relevant.

There are fundamental principles and rules that influence aggression, these rules are formed by the ideas of operant and response conditioning [4]. From this perspective, music functions as an important environmental stimulus that shapes emotional experience and psychological states [5]. It has been reported that music can evoke strong emotional responses, which vary depending on the type of music, the listening context, and individual listener characteristics [6]. Further studies investigated such impact and studied the frequent and extensive exposure of violent music genres in daily life, where it confirmed direct association between music that contains violent lyrics or media and negative behaviours such as violence, aggression, and substance abuse [7]. Anderson et al. also found that college students who listened to music categories with aggressive tone of music irrespective of the lyrics used, showed high levels of aggression when compared to music categories without aggressive tone or lyrics [7]. This highlights the psychological effects of music on university students and young people, where their choice of the kind of music they listen to could affect their emotions, social traits, behavioural responses and the shaping of their identities [7, 8].

Genres like hip- hop, rock, and pop often depict substance use as a way of escapism, status or to show rebellion, which normalize substance use and minimize the risk accompanied with its usage [9]. Such representation encourages the feeling of experimenting, especially among susceptible listeners viewing artists as their role models [10]. On the other hand, music was found to be a supportive component in addiction intervention and recovery contexts. Especially, when artists themselves share their personal experience with addiction and substance use, highlighting its harmful effects and encouraging their fans towards sobriety [11]. Also, studies showed that using music as a form of therapy in substance use disorders enhanced patient’s motivation towards treatment, improved their emotional wellbeing and reduced anxiety [12].

In addition to shaping behaviour, music interacts with psychological processes making traits such as perfectionism important to investigate. The conceptualisation of perfectionism has evolved over the past century. Early theoretical perspectives conceptualized perfectionism as a motivational drive toward mastery, while later clinical models emphasized its maladaptive aspects, including rigid self-standards, excessive self-criticism, and dependence on external validation [13–17]. Contemporary frameworks distinguish between adaptive and maladaptive dimensions of perfectionism, with the latter being associated with increased vulnerability to stress, anxiety, and emotional distress when performance expectations are not met [18]. Collectively, these interpretations establish perfectionism as a multifaceted personality trait characterized by heightened self-evaluation, sensitivity to approval or criticism, and vulnerability to emotional distress under perceived failure. Because music often serves as a tool for emotional regulation, self-reflection, and identity expression in young adults, perfectionistic tendencies may influence how individuals interpret and respond to music experiences, potentially amplifying either the stress-relieving or stress-enhancing effects of music engagement.

In educational contexts that involve music performance, perfectionism can exert an even stronger impact. Music students and performers face constant evaluation and high expectations, making them more susceptible to anxiety and stress when perfectionistic concerns are elevated [19–21]. Conversely, setting high but realistic standards can foster resilience, persistence, and confidence [22]. Recent evidence indicates that music students report higher levels of perfectionism and related anxiety than students in other disciplines, suggesting that music-related environments may amplify both the positive and negative dimensions of perfectionism [23]. Together, these findings highlight perfectionism as a key factor in understanding the behavioural and emotional effects of music among university students.

Furthermore, gender differences have been documented in both perfectionism and music engagement. Prior studies suggest that male students tend to internalize socially prescribed expectations more rigidly and demonstrate higher levels of narcissistic perfectionism traits, including hypercriticism, entitlement, and grandiosity [24–28]. These gendered psychological patterns may contribute to differences in music-related behaviours and risk profiles, particularly in relation to substance use. Therefore, exploring gender as a comparative factor is essential for understanding potential variability in the psychological effects of music among college students.

Despite a growing international literature, there remains a gap in understanding how music preferences intersect with psychological traits such as aggression, perfectionism, and substance use in non-Western youth populations. Egypt, with its rapidly shifting cultural norms and increasing exposure to global music trends, presents a unique setting to explore these dynamics.

This study aims to investigate the associations between music preferences, listening purposes, perfectionism dimensions, and substance use tendencies among Egyptian college students. Which is relevant to psychologists, counsellors, educators, and parents involved in supporting young adults during a critical developmental period. Additionally, the study provides a preliminary framework for researchers seeking to extend this work through more nuanced, longitudinal, or multivariate investigations into music-related behavioural and psychological outcomes.

### Research hypotheses

H1: Preference for rap and Egyptian folk music in social contexts (e.g., with friends or at parties) will be positively associated with substance use tendencies among emerging adults.

H2: Preference for classical Arab music will be negatively associated with substance use tendencies.

H3: Male students will report higher levels of narcissistic and self-critical perfectionism compared with female students.

These hypotheses guide the current investigation into the behavioural implications of music engagement among Egyptian emerging adults.

## Methods

### Procedure and participation

The study was approved by the Ethical Committee on Activities involving Human Subjects at the British University in Egypt and we followed the declaration of Helsinki principles. A total of 400 students between the ages of 18–24 years participated in this study after providing a consent with a response rate of 80%. However, 81 responses were excluded from the final analyses due to incomplete questionnaires, missing data, inconsistent reporting, or failure to meet the age inclusion criterion (≥ 18 years). Therefore, the final analysed sample consisted of 319 participants.

### Sampling and data collection

High school and college students from various Egyptian schools and universities were recruited to participate in this study. The data were collected using self-administered face to face questionnaires. In addition to, online questionnaires that were posted and shared via various social media platforms, including Facebook groups, Instagram stories, and WhatsApp family group chats. Participants were directed to a consent form with a description of the study and to ensure confidentiality. The study sample matched the typical age range of students, only individuals aged 18–24 was included. Survey responses were collected during the daytime after exams, between 11:00 a.m. and 3:00 p.m., at various campuses locations, including libraries, study lounges, classrooms, cafeterias, student centers, and computer labs. In the afternoon, responses were also collected in off-campus environments such as workspaces, coffee shops, and community centers. Nonetheless, because participation was voluntary and recruitment was partially online, some self-selection bias may have occurred and should be considered when interpreting the findings.

## Measures

### Music Listening

A revised version of Music Preference Questionnaire (MPQ-R) was used to assess music listening habits developed by Nater and colleagues [29]. In this study, we have used the English version in reference to Linneman et al., as English is the primary language of instruction in many Egyptian universities and most students in this age group are proficient English speakers. To enhance cultural relevance, the questionnaire was reviewed by bilingual researchers and contextually adapted with respect to music genre categories and listening contexts, while retaining the original structure and response format. As full psychometric validation of the MPQ-R within the Egyptian context was beyond the scope of the present study, the instrument was applied as an exploratory descriptive measure. Future research is warranted to establish its cross-cultural validity and psychometric properties.

Respondents indicated on a 5-point Likert scale, how often they listened to music, ranging from 1 (“Not at all”) to 5 (“Very much”) [30]. Then participants were provided with a list including five music categories and were asked to indicate the genres they often listened to using (yes/no) responses in accordance with Rentfrow and Gosling [31]. The genres provided included alternative pop, rap, classical Arab music, soul-funk, and modern Egyptian folk music. Further, the frequency of music listening was also requested, ranging from “half an hour” to “three hours or more” daily according to Paping and others [32]. Finally, participants were asked about the purpose of listening to music (e.g., for relaxation, activation, distraction, reducing aggression, evoking emotions, combating boredom or loneliness, or general enjoyment), as well as listening contexts (e.g., alone, with friends, background music, or at live parties) as demonstrated by Lonsdale and his team [33]. Music importance was rated on a 5-point scale.

### Drug use assessment

Drug use was evaluated through a two-stage process. Participants were first asked whether they had ever used drugs. Those who responded affirmatively were then presented with a list of specific substances (e.g., cannabis, LSD, Shabo, Voodoo, cocaine, Xanax) and asked to indicate which they had used. This approach is consistent with both international standards, such as the Monitoring the Future survey performed by Johnston et al, and regionally validated studies in Egypt following Kabbash and other, allowing for reliable assessment of both general and substance-specific drug use patterns [34, 35]. For the purposes of this study, substance use was operationalized as a binary variable (ever vs. never use) to capture general substance use tendency rather than frequency, recency, or severity, consistent with the exploratory objectives of the investigation.

### Perfectionism

To measure perfectionism, the Big Three Perfectionism Scale (BTPS) was used, a 45-item self-report instrument developed by Smith et al. [36] that assesses three higher-order dimensions: narcissistic perfectionism, self-critical perfectionism, and rigid perfectionism. Each dimension comprises several lower-order facets. In the present study, analyses were conducted at the subscale level, including socially prescribed perfectionism, self-criticism, doubts about action, concern over mistakes, other-oriented perfectionism, hypercriticism, entitlement, and grandiosity. Items were rated on a 5-point Likert scale ranging from 1 (“Strongly disagree”) to 5 (“Strongly agree”). Internal consistency reliability of the BTPS total scale and subscales was evaluated in the present sample using Cronbach’s alpha coefficients.

### Data analysis

Descriptive statistics such as frequencies and percentages were used to summarize the main study variables. Group comparisons for continuous variables were performed using Student’s t-test for independent samples, while chi-squared tests (χ²) were applied for categorical variables. Music genre preferences and listening contexts were treated as independent variables, while substance use tendency was analysed as the primary dependent variable in accordance with the stated hypotheses. Given the exploratory nature of the study and the categorical structure of the primary variables, bivariate analyses were selected to identify initial associations between music preferences, perfectionism traits, and substance use tendencies. Multivariate adjustment for potential confounders was beyond the scope of this initial investigation and is recommended for future research. All statistical analyses were conducted using IBM SPSS Statistics for Windows, Version 20.0 (IBM Corp., Armonk, NY, USA). A p-value < 0.05 was considered statistically significant. All categorical variables (e.g., music genres, listening contexts) were analysed as separate dimensions without assuming ordinal or linear relationships among categories.

## Results

### Sample characteristics

The study sample included 319 participants, of whom 40.8% were male. Ages ranged from 18 to 24 years. A total of 46 participants (14.4%) reported drinking, smoking, or using illicit drugs in the past 12 months. Marijuana use was reported by 27 students (8.5%), while 7 students (2.2%) reported club drug use (Table 1).

**Table 1:** Demographic characteristics of sample population (*N* = 319)

Demographic Variable	Category	*N*	Percentage %
Gender	Male	130	40.8%
	Female	189	59.2%
Age Group (years)	18	30	9.4%
	19–20	74	23.2%
	21–22	62	19.4%
	23–24	153	47.9%
Education Level	Undergraduate	313	98.1%
	Graduate	6	1.9%
Field of Study*	Pharmacy	106	33.2%
	Engineering	47	14.7%
	Business	28	8.8%
	Arts & Design	27	8.5%
	Mass Communication	15	4.8%
	Psychology	10	3.2%
	Other†	86	26.9%

* Field of Study: grouped by frequency for clarity

† Includes Law, Dentistry, Biotechnology, Medicine, Nursing, Physiotherapy, Nutrition, Translation, Political Science, Computer Science and Tour Guide

### Music listening

Sixty-three respondents (19.7%) reported listening to music “daily for 30 minutes or less”. The most frequently reported music genres were Classical Arab Music (69.3%), Rap (67.1%), Soul/Funk (56.2%), Egyptian Folk Music (55.5%), and Pop (35.2%), respectively (Table 2).

**Table 2:** Information about music genres and the frequency of listening

Music Genre	Does not Listen		Listens	
	Frequency (f)	Percentage (%)	Frequency (f)	Percentage (%)
Pop	207	64.8	112	35.2
Rap	105	32.9	214	67.1
Classical Music	98	30.7	221	69.3
Soul/Funk	140	43.8	179	56.2
Egyptian Folk Music	142	44.5	177	55.5

### Findings

To test H1, chi-squared tests were used to examine musical preferences differences in students by gender and substance use tendencies. As shown in Table 3, a significant association was observed between gender and preference for rap music, χ² (1, *N* = 319) = 21.76, *p* < 0.001, with a higher proportion of male students reporting listening to Rap music compared to female students. In addition, the listening context for Rap music differed significantly by substance use tendency, χ² (1, *N* = 319) = 9.34, *p* = 0.002. Students with a substance use tendency were more likely to listen to Rap music in social settings (e.g., with friends or at live parties) than those without such a tendency (Table 4).

**Table 3:** Association between Music Preferences and Substance Use Tendency

Music Genre	Substance Use: Yes (*n*, %)	Substance Use: No (*n*, %)	χ²	df	*p*-value
Rap	63 (65.0%)	146 (65.7%)	0.19	1	0.88
Classical Arab Music	59 (61.5%)	164 (73.5%)	4.65	1	0.03*
Egyptian Folk Music	65 (67.7%)	84 (37.7%)	24.3	1	< 0.001*
Pop	68 (70.8%)	143 (62.7%)	1.34	1	0.24
Soul/Funk	59 (60.8%)	114 (51.4%)	2.44	1	0.12

**Table 4:** Findings Related to Differences in Music Preferences Listening Situation and Tendency to Use Substances

Music Genre	Listening Context	Substance Use: Yes (*n*, %)	Substance Use: No (*n*, %)	χ²	df	*p*-value
Rap	Alone / Background	28 (45.2%)	170 (66.1%)	9.34	1	0.002*
	With Friends / Live Parties	34 (54.8%)	87 (33.9%)			
Classical Arab Music	Alone / Background	22 (51.2%)	172 (60.4%)	1.3	1	0.25
	With Friends / Live Parties	21 (48.8%)	113 (39.6%)			
Egyptian Folk Music	Alone / Background	21 (51.2%)	130 (55.1%)	0.21	1	0.64
	With Friends / Live Parties	20 (48.8%)	106 (44.9%)			

Further analyses revealed a significant relationship between preference for Classical Arab music and substance use tendency, χ² (1, *N* = 319) = 4.65, *p* = 0.03. Consistent with H2, students who did not show tendency toward substance use were more likely to listen to classical Arab music when compared to those with substance use tendency. Egyptian Folk music preference was also significantly associated with substance use tendency, χ² (1, *N* = 319) = 24.30, *p* < 0.001, with higher rates of substance use reported among those who preferred Egyptian Folk music compared to those who did not (Table 3). However, no significant associations were found between music listening purpose and substance use tendency for Rap music, Classical Arab music, or Egyptian Folk music (Table 5).

**Table 5:** Findings Related to Differences in Music Listening Purpose and Tendency to Use Substances

		**Tendency Towards Drug Abuse**				**X2**	**df**	***p*** **-value**
		**Existent**		**Non-Existent**				
		*N*	%	*N*	%			
Rap	To reduce aggression	20	40.8	95	40.6	0.0008	1	0.97
	To increase certain feelings	29	59.2	139	59.4			
Classical Arab Music	To reduce aggression	16	42.1	101	66.5	0.068	1	0.79
	To increase certain feelings	22	57.9	152	33.5			
Egyptian Folk Music	To reduce aggression	19	16.7	83	83	0.00012	1	0.99
	To increase certain feelings	95	83.3	17	17			

Prior to inferential analyses, internal consistency reliability of the Big Three Perfectionism Scale (BTPS) was evaluated. The total scale demonstrated excellent internal consistency (Cronbach’s α = 0.93). Subscale reliability coefficients ranged from acceptable to good, including socially prescribed perfectionism (α = 0.74), self-criticism (α = 0.84), other-oriented perfectionism (α = 0.84), narcissistic perfectionism facets (α = 0.80), and rigid perfectionism (α = 0.70). These findings indicate adequate internal consistency for all perfectionism variables used in subsequent analyses.

To test H3, independent samples t-test assessed gender differences in perfectionism scores. Male students scored significantly higher than female students on the socially prescribed perfectionism subcomponent of self-critical perfectionism, t (256) = 2.12, *p* = 0.028 (Table 6). Regarding narcissistic perfectionism, males scored significantly higher than females across all subcomponents, including other-oriented perfectionism, hypercriticism, entitlement, and grandiosity (Table 7).

**Table 6:** Comparisons of different components of self-critical perfectionism scores according to gender

	Cronbach’s α	Male M(SD)	Female M(SD)	t(df = 256)	*p*-value
Socially Prescribed Perfectionism	0.74	11.78 (3.33)	10.92 (3.57)	2.21	0.028*
Self-criticism	0.84	12.01 (3.75)	11.88 (4.32)	0.16	0.876
Doubts About Action	0.84	13.91 (4.25)	13.76 (4.82)	0.23	0.825
Concern Over Mistakes	0.84	14.54 (4.07)	14.61 (4.81)	0.09	0.928

For quantitative variables, M (SD); for categorical variables, *n* (%)

**Table 7:** Comparisons of different components of narcissistic perfectionism scores according to gender

	Cronbach’s α	Male M(SD)	Female M(SD)	t(df = 256)	*p*-value
Other-Oriented Perfectionism	0.84	12.82 (4.78)	11.62 (4.13)	2.11	0.035*
Hypercriticism	0.80	9.95 (3.29)	9.09 (3.31)	1.99	0.047*
Entitlement	0.80	10.71 (3.63)	9.63 (3.31)	2.39	0.017*
Grandiosity	0.80	11.81 (3.51)	10.34 (2.96)	3.54	<0.001**

For quantitative variables, M (SD); for categorical variables, *n* (%)

## Discussion

Recent neuropsychological research provides a useful contextual framework for understanding behavioural patterns associated with narcissistic perfectionism. Previous studies have reported that substance use is associated with alterations in brain rhythms, particularly within frontal regions involved in executive functions such as impulse control, behavioural inhibition, and decision-making. Disruptions in these neural systems have been linked to increased impulsivity and risk-taking behaviours in individuals with substance use problems. While such neurobiological models have been proposed in prior research, the present study focused exclusively on behavioural and self-report data; neurobiological findings are therefore referenced only to provide theoretical context, without implying underlying neural mechanisms in the current sample [37, 38].

Our findings demonstrated an association between preference for Egyptian folk music and a higher tendency toward substance use. Previous EEG research has reported that altered resting-state brainwave activity, particularly within frontal cortical regions, may be associated with increased vulnerability to addictive behaviours. These neurophysiological findings are referenced here solely to provide theoretical context and may help explain heightened susceptibility observed in some populations, without implying underlying neural mechanisms in the present sample [39]. In contrast, classical music has been shown to induce EEG patterns indicative of a relaxed state, characterized by reduced arousal and attentional engagement. Accordingly, the observed association between classical music preference and substance use tendency in the present study is more likely to reflect broader psychosocial or affective processes, rather than direct neurobiological effects [39]. Overall, these findings suggest that different auditory environments may be linked to substance use tendencies through complex psychological and contextual pathways that needs further investigation.

Rap music preference, particularly when experienced in social settings such as live events or peer gatherings, was also associated with greater substance use tendency. This observation aligns with existing literature indicating that rap music frequently contains references to substance use, and that exposure to such content may coincide with environments where substance use is more socially normative [40]. Large-scale surveys have similarly reported associations between preferences for rap or hip-hop music and higher rates of smoking, drinking, and illicit substance use, even after controlling for demographic factors [41, 42]. Importantly, these associations do not imply that music exposure causes substance use, but rather that music preferences may cluster with certain social contexts, identity expressions, and behavioural patterns.

The interaction between high arousal musical environments, peer influence, and personality traits such as narcissistic perfectionism may be particularly relevant in understanding these patterns. Individuals high in narcissistic perfectionism often seek external validation and may be more inclined toward sensation seeking or socially reinforced behaviours [43]. However, given the cross-sectional nature of the present study, the directionality of these relationships cannot be determined. It is equally plausible that engagement in substance use shapes music preferences, as psychoactive experiences, altered affective states, and peer group norms may influence the types of music individuals choose to listen to. Longitudinal studies are therefore needed to clarify whether music preference precedes substance use, results from it, or develops alongside it within shared social contexts.

Together, these findings illustrate a complex interaction between cultural influences, personality traits, and behavioural tendencies associated with substance use, rather than a unidirectional causal pathway.

Regarding gender differences, male students scored significantly higher than female students on socially prescribed perfectionism, a key component of self-critical perfectionism. This finding is consistent with prior research suggesting that males may internalize external expectations more rigidly, particularly in competitive academic or social environments, which may contribute to elevated socially prescribed perfectionism scores [24, 44].

Moreover, males scored significantly higher across all measured dimensions of narcissistic perfectionism, including other-oriented perfectionism, hypercriticism, entitlement, and grandiosity. Men scored higher in other-oriented perfectionism (*p* = 0.035), suggesting a greater propensity to impose elevated standards on others rather than themselves. This aligns with prior research showing that perfectionism often manifests outwardly as controlling and critical interpersonal behaviour [25, 45].

Similarly, men exhibited significantly greater hypercriticism (*p* = 0.047), which may reflect social norms emphasizing competitiveness and emotional restraint, where criticism serves as a mechanism for maintaining status and enforcing external standards [26, 46].

Entitlement was also significantly higher among men (*p* = 0.017), reflecting stronger beliefs about deserving special treatment and superiority. This finding is consistent with literature linking masculine identity to dominance, achievement, and self-enhancement [27, 47].

Grandiosity showed the most robust gender difference (*p* < 0.001), with men reporting markedly greater inflated self-importance and desire for admiration. This trait can motivate unrealistic standard setting not only for self-fulfilment but also for validating an idealized self-image, often causing interpersonal tension when expectations are unmet [28].

Collectively, these gender differences support the conceptualization of narcissistic perfectionism as a multifaceted construct shaped by sociocultural factors. Where, masculine identity is often closely tied to achievement, public recognition, and social status, which may reinforce outwardly directed perfectionism and entitlement. In contrast, women may be more likely to internalize perfectionistic standards, resulting in greater self-critical tendencies [48–51].

From a broader international perspective, comparable associations between music preference, personality traits, and substance use have been documented across diverse cultural contexts. Studies from North America and Europe have consistently linked preferences for high arousal genres, such as rap and electronic music, with sensation seeking and greater substance use tendencies [52, 53]. Conversely, slower or more structured genres, including classical and traditional forms, have been associated with emotion regulation and stress management motives [54]. Moreover, cross-cultural investigations demonstrate that music preferences reflect universal psychological needs shaped by cultural values [55].

Together, these findings situate the present results within a global framework that recognizes both shared and culture specific pathways linking music engagement, perfectionism, and behavioural outcomes.

## Limitations

Despite the relevance of these findings, the study relied exclusively on self-report questionnaires, which may introduce recall bias and social desirability bias, particularly regarding sensitive behaviours such as substance use. Although anonymity and confidentiality were emphasized, underreporting cannot be ruled out. Future research may benefit from incorporating multimethod assessments (e.g., peer reports, behavioural measures) or follow-up interviews to improve accuracy and reduce bias in measuring substance use tendencies. In addition, substance use was assessed as a lifetime binary indicator and did not capture frequency, recency, or severity of use, which limits differentiation between experimental, occasional, and problematic substance use.

Additionally, the predefined genre categories may have restricted the representation of participant’s full music preferences, as emerging hybrid music trends and niche subgenres were not assessed. Future research may incorporate open-ended or more granular genre categorizations to better reflect the rapidly evolving music landscape.

Furthermore, the study did not control for potential confounding variables such as socioeconomic status, academic stress, or mental health symptoms, which may influence both music preferences and substance use risk. Future studies incorporating multivariate statistical models are recommended to better isolate the unique contributions of music engagement to behavioural outcomes.

Finally, the distribution of substance use tendency was not balanced across gender groups, which may have influenced the observed associations. This imbalance limits the generalizability of gender-based comparisons and may reduce statistical power for subgroup analyses. Future studies with more balanced gender representation or stratified analytical approaches are recommended.

## Conclusion

This study highlights significant associations between music preferences, listening purposes, perfectionism traits, and substance use tendencies among Egyptian college students. Our findings suggest gender difference in music preference, with male students showing a stronger preference for rap music particularly in social settings associated with higher substance use tendency.

Egyptian folk music was also linked to increased substance use, potentially reflecting underlying neurological patterns related to reduced frontal activation. Conversely, classical Arab music showed an inverse association with substance use.

Further, male students exhibited higher scores in both socially prescribed and narcissistic perfectionism, indicating gendered patterns in perfectionistic traits that may be embedded within broader personality features. These results underscore the importance of music based behavioural insights into public health strategies, particularly for substance use and youth mental health.

## Data Availability

The datasets generated and/or analyzed during the current study are not publicly available due to sensitivity and privacy considerations. The data are available from the corresponding author upon reasonable request.

## Abbreviations

MPQ-R: Music preference questionnaire
BTPS: Big three perfectionism scale
QEEG: Quantitative electroencephalography
